# From fear to fascination: transforming interest in interventional radiology

**DOI:** 10.1007/s00330-024-10901-6

**Published:** 2024-07-09

**Authors:** Elif Can

**Affiliations:** Department of Diagnostic and Interventional Radiology, Medical Center University of Freiburg, Faculty of Medicine, University of Freiburg, Hugstetter Str. 55, 79106 Freiburg, Germany

Interventional Radiology (IR) stands at the intersection of rapid technological advancements and a pressing need for specialized professionals. The evolving landscape of IR, with its intricate procedures and potential for patient care innovation, presents both an opportunity and a challenge. The critical question is: How can formal education and clinical exposure mitigate students’ doubts and foster their interest in IR?

In this issue of European Radiology, the study by Cao et al addresses this question head-on, revealing the profound impact of targeted educational interventions [1]. Their findings are compelling: structured exposure to IR curricula and clinical settings significantly reduces students’ anxiety about radiation, a longstanding barrier to entering the field. This reduction in fear is crucial for demystifying IR and making it a more attractive career option.

Moreover, the study highlights that hands-on clinical experiences play a pivotal role in sparking genuine interest and enthusiasm for IR. Students who participated in these experiences showed a marked increase in considering IR as a career. This underscores the importance of experiential learning in medical education, particularly for fields that are often perceived as daunting or highly technical.

However, an important issue that the study uncovers is the gender disparity in career choice post-intervention. While both male and female students exhibited reduced anxiety and increased interest, male students were more likely to pursue IR careers. This disparity is concerning, especially as the medical field sees a growing number of female graduates. Addressing this gap requires targeted efforts to engage and support female students, ensuring equal representation in all medical specialties, including IR [2, 3].

Looking ahead, the implications of these findings are significant for the future of medical education and the IR workforce. Integrating robust IR modules into medical school curricula, which combine theoretical knowledge with practical, hands-on experiences, can help mitigate unfounded fears and ignite interest in the field. In addition, mentor- and sponsorship programs and supportive networks for female students can bridge the gender gap, fostering a diverse and inclusive IR community [2, 4, 5].

The study’s insights resonate with the broader educational theories presented in the ICAP Framework by Chi and Wylie, which emphasize the importance of interactive and constructive engagement for deeper learning outcomes [6]. This framework supports the idea that deeper engagement through real-world experiences leads to better understanding and retention of knowledge.

In conclusion, the study by Cao et al provides a roadmap for enhancing the appeal of interventional radiology among medical students [1]. By leveraging these insights to develop comprehensive educational programs that reduce anxiety, foster interest, and encourage diverse career considerations, we can ensure a steady pipeline of skilled and enthusiastic professionals ready to advance the field of interventional radiology.
